# Towards human central nervous system *in vitro* models for preclinical research: strategies for 3D neural cell culture

**DOI:** 10.1186/1753-6561-5-S8-P53

**Published:** 2011-11-22

**Authors:** Daniel Simão, Inês Costa, Margarida Serra, Johannes Schwarz, Catarina Brito, Paula M Alves

**Affiliations:** 1 Instituto de Tecnologia Química e Biológica –Universidade Nova de Lisboa, 2780-157 Oeiras, Portugal; 2 Instituto de Biologia Experimental e Tecnológica, 2780-901 Oeiras, Portugal; 3 Department of Neurology, University of Leipzig, 04103 Leipzig, Germany

## Background

The development of new drugs for human Central Nervous System (CNS) diseases has traditionally relied on 2D *in vitro* cell models and genetically engineered animal models. However, those models often diverge considerably from that of human phenotype (anatomical, developmental and biochemical differences) [1] contributing to a high attrition rate - only 8% of CNS drugs entering clinical trials end up being approved [2]. Human 3D *in vitro* models are useful complementary tools towards more accurate evaluation of drug candidates in pre-clinical stages, as they present an intermediate degree of complexity in terms of cell-cell and cell-matrix interactions, between the traditional 2D monolayer culture conditions and the complex brain and can be a better starting point for the analysis of the *in vivo* context. Aiming at developing novel 3D *in vitro* models of the CNS, this work focus on the implementation of long-term cultures of human midbrain-derived neural stem cells (hmNSC) for the scalable supply of neural-subtype cells, with a focus on the dopaminergic lineage, following a systematic technological approach based on stirred culture systems.

## Materials and methods

Cell culture: hmNSC were isolated as previously reported [3] and routinely propagated in static conditions, on poly-L-ornithine-fibronectin (PLOF) coated plates, in serum-free propagation medium, containing basic fibroblast growth factor and epidermal growth factor [3]. hmNSC were cultured in stirred systems in Cultispher S microcarriers (Percell Biolytica) without coating and coated with PLOF) or as neurospheres for 7 to 21 days, with media changes every 3-4 days. All experiments were performed in 125 mL shake flasks (20 mL working volume), with orbital shaking at 100 rpm. Cultures were maintained at 37°C, in 3% O_2_. Double stain viability test: aggregates were collected from stirred cultures, incubated with fluorescein diacetate (10 μg/mL) and propidium iodide (1 μg/mL) and observed on a fluorescence microscope (Leica DMI6000). Aggregate size was measured in pictures taken from each culture sample using Image J software (NIH), as previously reported [4]. Dissociation: For microcarrier cultures, Cultispher S was allowed to settle, washed with PBS and digested with Trypsin 0.05%-EDTA (Gibco). Cells were collected by centrifugation and counted by trypan blue exclusion dye. Free cells were counted using the same aliquot. Aggregates were dissociated with Accutase (Sigma).

## Results

**Figure 1:**
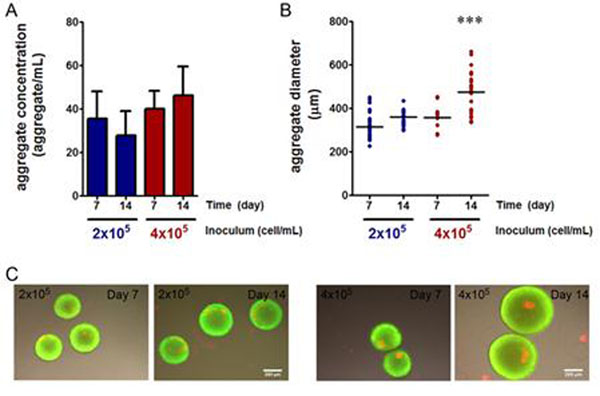
Effect of inoculum concentration on aggregate concentration (A), aggregate size (B) and viability (C), evaluated using a double stain viability test: fluorescein diacetate (live, green); propidium iodide (dead, red). Cells were cultured in shake flasks with inoculum concentrations of 2 and 4x10^5^ cell/mL. Error bars denote standard deviation of average of 3 independent experiments. *** indicates significant difference (P<0.001) in aggregate diameter by one-way ANOVA analysis with a Tukey’s post-hoc multiple comparison test.

The feasibility of culturing hmNSC as 3D structures in stirred culture systems was assessed by testing two different approaches: microcarrier technology versus cell aggregated cultures (neurospheres). For the first strategy, Cultispher S, a collagen-based macroporous microcarrier was tested for its ability to support hmNSC attachment and growth. Microcarriers uncoated and coated with PLOF were tested. hmNSCs were labelled with PKH26 lipophilic dye (red) for detection purposes and inoculated at 1.5x10^5^ cell/mL, in a carrier concentration of 1g/L, corresponding to approximately 125 cell/microcarrier. Monitoring along 5 days of culture time revealed that the fraction of viable cells found on the microcarriers was less than 10% of the inoculum, for both uncoated and PLOF-coated microcarriers, indicating poor microcarrier colonization. Fluorescence microscopy analysis revealed that hmNSC aggregated in suspension rather than colonizing Cultispher S microcarriers (not shown). For the cell aggregate strategy, two inoculum concentrations were tested - 2 and 4x10^5^ cell/mL and aggregate size and number evaluated along culture time (Figure 1).

The inoculum concentration of 2x10^5^ cell/mL was as efficient as 4x10^5^ cell/mL in promoting cell aggregation (Figure 1A) whereas it allowed for lower mean diameters along culture time (362±32 μm at day 14) as compared to the higher inoculum concentration for which significantly higher mean diameter and also a wider range of aggregate sizes were observed (475±103 μm at day 14) (Figure 1B). Moreover, the lower inoculum concentration avoided the formation of necrotic centres, which were detected in cultures with an inoculum concentration of 4x10^5^ cell/mL (Figure 1C).Taken together the data presented indicates that 2x10^5^ cell/mL is the most favourable inoculum concentration for culture of hmNSC as aggregates in stirred culture systems.

## Conclusions

In this study the feasibility of culturing hmNSC as 3D structures in stirred culture systems was evaluated. Cell aggregates (neurosphere) culture, using an inoculum concentration of 2x10^5^ cell/mL was selected as the best strategy, due to the higher cell viabilities and tightly control of aggregate diameter attained. The implemented 3D culture system will be applied in the optimization of differentiation of hmNSC into dopaminergic neurons, astrocytes and oligodendrocytes.
